# The Royal Portsmouth, Portsea, and Gosport Hospital

**Published:** 1892-06-25

**Authors:** 


### The Institutional Workshop.

### WITHIN THE HOSPITALS.

### III.—THE ROYAL PORTSMOUTH, PORTSEA, AND GOSPORT HOSPITAL.

This institution, founded about 45 years ago, presents several features of interest. The original buildings are old, and so many additions have been made from time to time that the whole of them now must be classed as belonging to the irregular or "heap of buildings" type. Hospital buildings erected under these circumstances necessitate an unusual amount of corridor space, which adds not a little to the cost and difficulty of administration. It must be very tiring, for instance, for the Matron to make her daily inspection of this hospital. Six years ago the whole institution was in a state which left much to be desired. The sanitary arrangements, the nursing, and even the food were unsatisfactory. At the present time, however, owing to the devoted labours of the present Matron, Miss Tillett, and to the continuous and intelligent support of the Committee, which has had the wisdom to vote the necessary funds, all, or nearly all, that was objectionable has been removed. The hospital bears throughout the stamp of being well administered in every department, and we cannot but feel that the Committee is to be congratulated on having at the head of its affairs so able and devoted a lady as Miss Tillett undoubtedly is. No one with a knowledge of hospitals can now visit the wards of the Royal Portsmouth Hospital without being impressed with a feeling of admiration for the Matron, as everything bears the aspect of good management throughout her department.

#### The Wards.

This hospital contains some excellent wards, many of which are single storey pavilions. The floors are polished, the lavatories and bath-rooms are in good order, with tiled walls, and the ward kitchens contain some of the best American stoves we have seen anywhere. Although we visited the hospital at an inconvenient hour, and paid a surprise visit, we found everything in good order and beautifully clean. The nurses seemed to be carefully trained (some of their bandaging would have done credit to the house-surgeon of a large metropolitan hospital), and the patients are well cared for and most comfortable. This is high praise, but it is well deserved, especially as we know from experience the difficulties of maintaining an old building like this at the high pitch of administrative excellence which it has undoubtedly attained. In all directions evidence was forthcoming of the interest displayed by all in the well-being of the institution, and in small details like the crockery for the patients' use, and the condition of the ward-lockers and cupboards, little if anything, was left to be desired. Although the difficulty of providing such accommodation must have been serious, each ward has a separate room in which the patients' clothes are classified and arranged, and each of the head nurses is now provided with a small room, in which she can sit in comfort.

#### The Children's Wards.

These wards are a great feature of the Royal Portsmouth Hospital. They are fine, airy, well-arranged, and beautifully kept. One of them is devoted to boys, and the other to girls. A head nurse has charge of both, and under her is a charge nurse and two probationers. The children are evidently happy and well-cared for, and we note with pleasure that the townspeople take a good deal of interest in their welfare. The two children's wards are separated by a nurse's room, bath-room, linen store-room, and ward kitchen. We were glad to notice that Rufford's baths had been fixed in this ward, a fact which proves the existence of a desire on the part of the Managers to keep well apace with recent improvements. We visited these wards in the morning, and noticed an absence of toys. The reason given for this was that all toys are taken out of the wards in the evening, and placed in the toy-room until after the doctors' morning visits. This is an excellent arrangement, and adds much to the happiness of the children, as it causes them to place a much higher value on the toys than if they had them always in the ward. Besides, it enables the Sisters to arrange that each child shall have a variety of playthings from day to day.

#### The Site, Gardens, and Grounds.

The site is well adapted to its purpose and there is an abundance of garden space. The kitchen garden is amply sufficient to provide the hospital with fresh vegetables, and we were pleased to notice that one portion of it adjacent to the nurses' tennis ground, was set apart as a flower garden for the Matron. This is the second Matron's garden that we have come across in the course of our inspections during the last few months, and it makes us suggest that London hospital committees might beneficially provide a small green-house or conservatory as an excellent means of relaxation and source of interest for the Matron. There can be no doubt that the continuous residence in a hospital entailed upon the Lady Superintendents and Matrons from the pressure of urgent and manifold duties often prevents these ladies from getting an adequate amount of exercise. The provision of a garden in connection with country, or a small conservatory in the case of metropolitan hospitals, would be a kindly and beneficial act, and we hope the practice will become universal. There must be many well-to-do Governors who would gladly provide the necessary means for erecting and stocking these conservatories, and we look forward to the time when no well-administered hospital will be without them. Flowers and ferns in the wards tend to promote the recovery of the patients by brightening their surroundings, and these conservatories would be very useful in helping to keep up a constant supply.

#### Isolation Blocks.

In the grounds are two separate buildings, one of which is used for the admission of cases which require quiet and special nursing, and the other for the reception of infectious and other cases which it is desirable to remove from the general wards. The former block is sometimes used for the admission of paying patients, for the rules provide that only really necessitous patients shall be admitted to free relief. No person receiving parochial relief, and no person in the receipt of 20s. a week with less than three children, can obtain relief except on payment of 2s. a day as an in-patient or such smaller sum as the visitors for the month may determine. There is one rule, however, which modern surgeons would scarcely approve of we fancy. It provides that no person suffering from any cancer not admitting of an operation can be treated as an in-patient.

#### The General Canvass.

A perusal of the report shows that all classes of people take a great interest in this hospital. It has been the practice, with a view to stimulating the inhabitants of the town and neighbourhood, to give still greater support, to organise a periodical canvass of the borough for increased annual subscriptions. The last of these canvasses was made in 1885, and resulted in the addition of £590 to the funds of the hospital. These canvasses are organised by a special committee, consisting of members of the Committee of Management and subscribers appointed at the annual meeting. The borough is divided into districts according to its municipal wards, one councillor and two members of the Local Board, who are willing to assist in the work, having the management of each district. Experience shows that ladies very willingly identify themselves with this movement and prove most excellent canvassers. A similar practice has long been carried out in connection with the Royal Infirmary, Edinburgh, where an annual house-to-house collection is made, which produces many hundreds of pounds. Would it not be possible for the metropolitan hospitals to institute a similar system of canvass, modified according to the special circumstances of each institution? The experiment, at any rate, is well worthy of a trial, and we hope some of the more enterprising of the metropolitan hospital committees will put it to the test of practice.

The Nursing and Domestic Arrangements.

The kitchen is well organised, and the hot-water tins in which the dinners are supplied to the patients struck us as being specially good. We noticed, however, in the meat store that the meat is at present purchased in carcase. Although no doubt the cost per pound is less where this custom prevails, experience proves that it leads to so much waste in various ways that the actual cost is, in the end, greater under this system than it is when tenders are made out for each joint separately. We would venture to suggest to the Committee that they would reduce the cost per bed and give the patients, on the whole, better food if they were to cease buying in carcase and to adopt the joint system. We should be very glad to supply them if desired with a model form of tender, and to explain how this latter system could best be carried out. The servants have a comfortable sitting-room to themselves, and the nurses' common-room is a fine apartment, and marks the advance which has taken place in the internal arrangements of this hospital in recent years.

We give a special account of the nursing in *The Mirror*, and need not, therefore, devote further space to it here.

